# Phylogenetic Reconstruction and DNA Barcoding for Closely Related Pine Moth Species (*Dendrolimus*) in China with Multiple Gene Markers

**DOI:** 10.1371/journal.pone.0032544

**Published:** 2012-04-03

**Authors:** Qing-Yan Dai, Qiang Gao, Chun-Sheng Wu, Douglas Chesters, Chao-Dong Zhu, Ai-Bing Zhang

**Affiliations:** 1 College of Life Sciences, Capital Normal University, Beijing, People’s Republic of China; 2 Key Laboratory of Zoological Systematics and Evolution, Institute of Zoology, Chinese Academy of Sciences, Beijing, People’s Republic of China; Pennsylvania State University, United States of America

## Abstract

Unlike distinct species, closely related species offer a great challenge for phylogeny reconstruction and species identification with DNA barcoding due to their often overlapping genetic variation. We tested a sibling species group of pine moth pests in China with a standard cytochrome c oxidase subunit I (COI) gene and two alternative internal transcribed spacer (ITS) genes (ITS1 and ITS2). Five different phylogenetic/DNA barcoding analysis methods (Maximum likelihood (ML)/Neighbor-joining (NJ), “best close match” (BCM), Minimum distance (MD), and BP-based method (BP)), representing commonly used methodology (tree-based and non-tree based) in the field, were applied to both single-gene and multiple-gene analyses. Our results demonstrated clear reciprocal species monophyly for three relatively distant related species, *Dendrolimus superans*, *D. houi*, *D. kikuchii*, as recovered by both single and multiple genes while the phylogenetic relationship of three closely related species, *D. punctatus*, *D. tabulaeformis*, *D. spectabilis*, could not be resolved with the traditional tree-building methods. Additionally, we find the standard COI barcode outperforms two nuclear ITS genes, whatever the methods used. On average, the COI barcode achieved a success rate of 94.10–97.40%, while ITS1 and ITS2 obtained a success rate of 64.70–81.60%, indicating ITS genes are less suitable for species identification in this case. We propose the use of an overall success rate of species identification that takes both sequencing success and assignation success into account, since species identification success rates with multiple-gene barcoding system were generally overestimated, especially by tree-based methods, where only successfully sequenced DNA sequences were used to construct a phylogenetic tree. Non-tree based methods, such as MD, BCM, and BP approaches, presented advantages over tree-based methods by reporting the overall success rates with statistical significance. In addition, our results indicate that the most closely related species *D. punctatus*, *D. tabulaeformis*, and *D. spectabilis*, may be still in the process of incomplete lineage sorting, with occasional hybridizations occurring among them.

## Introduction

DNA barcoding (http://www.barcodinglife.org) has gained widespread prominence during the past eight years as part of the worldwide campaign to develop a global biodiversity inventory [1]–[14]. On 23 Aug. 2011, there were 1,348,985 barcode records from 110,892 species in the Barcode of Life Database (BOLD) (www.barcodinglife.org). However, some reservations still remain about the utility of DNA barcoding [15]–[24]. Two main issues, the choice of barcoding gene and methods for species assignments, have been the central problems.

The choice of barcoding gene is one of the primary issues. The 5 prime segment of the mitochondrial (mt) cytochrome c oxidase subunit I (COI) gene (648 bp) was initially proposed to serve as DNA barcode [1]–[2], and proved to be of great success in many animal groups [1]–[2], [25]. Currently, COI has been selected as a standard barcode gene for animal groups. However, the rationale of selection of COI as standard barcode is subject to debate, and with the increase in barcoded taxa, from algae, fungi, bacteria and plants to invertebrates and vertebrates, scientists have found its less effective in some taxon groups [2], [20], [26]–[28]. The search for the most suitable gene for species identification is not over, with several recent studies testing the efficiencies of different genes, using part of, or the whole of mtDNA genome to look for the optimal DNA barcode gene [29]–[30]. On the other hand, empiricists have also proposed other gene segments as candidate DNA barcode loci, such as the nuclear ITS regions (ITS1, ITS2) [31]–[32]. ITS - Internal Transcribed Spacer (ribosomal DNA repeating unit), which is a commonly used DNA biomarker, was suggested and examined in several plant groups [31]–[32], and fungi (http://www.boldsystems.org/views/projectmenu.php?&). This widely used genetic marker might be suitable as a DNA barcode due to its highly variability. This is especially the case for groups composed of closely related species, where the rate of successful species identification with COI is relatively low (less than 70%) (e.g., fly, [20]). Unlike groups of distantly related species, where the existence of large genetic divergence between species makes discrimination easy, groups of closely related species offer greater challenges for phylogenetic reconstruction and clear species identification.

Pine moth species (caterpillar) are one of the most serious pest insect group in China [33]–[39], with outbreaks of the pest regularly causing extensive forest damage [34]–[35], [37]–[39]. This pest species group consists of six commonly occurring, closely related species, between which discrimination is very challenging. Taxonomically, three of them (*Dendrolims punctatus* [40], *D. tabulaeformis* [41], *D. spectabilis* [42]) have a very uncertain species status. For instance, the latter two had been suggested as a subspecies of *D. punctatus* [34]–[35]. However, these species were treated as three different species in several other studies [37]–[39]. Therefore, this species group provides a good model for investigating the efficiency of DNA barcode species identification for closely related species groups.

In addition to the selection of barcoding region, the methods used to assign a query to species in the reference database has been another hotly debated issue [1]–[2], [10], [12], [18], [20], [24], [43]–[50]. Several barcoding methods have been used or proposed in the current DNA barcoding campaign, including tree-based methods (ML, NJ), distance-based methods (the “best close match” (BCM), [20]), Bayesian methods [48]-[49], pure clustering methods [51], BP-based methods [12], [52], and the fuzzy-set-thoery-based method [53]. Five of these are selected (Maximum likelihood (ML)/Neighbor-joining (NJ), “best close match” (BCM), Minimum distance (MD), and BP-based method (BP)), as representatives of different types of methods, to apply in current study. Apart from the tree-based methods, we performed 14710 simulation replicates, analyzing the genes individually or in combination. The main goal of this study is to examine the phylogenetic relationship among those closely related species, and the second is to compare the performance of the standard COI gene, the nuclear rDNA genes ITS1 and ITS2, and their combinations in identification of closely related pine moth species in this study. In addition, we factor in the success rate of DNA sequencing. A successful species identification with a given DNA barcoding system includes several steps: genomic DNA extraction, PCR, sequencing, and species assignments. The success rate and accuracy of the former two steps (DNA extraction, PCR) in a DNA barcoding system have been documented [1]–[2], [43], in particular in the use of museum collections [54]. However, the effect of sequencing success rates on DNA barcoding is remarkably ignored in most current studies. Therefore, we also propose that the success rate of a DNA barcode system takes into account both sequencing success and assignment success (different barcoding methods/algorithms), since some potential DNA barcoding markers, such as ITS, may suffer from low sequencing success rates due to heterogeneity of different ITS copies in the same individuals.

## Results

### Phylogenetic Inferences and Haplotype Network Reconstruction

**Figure 1:**
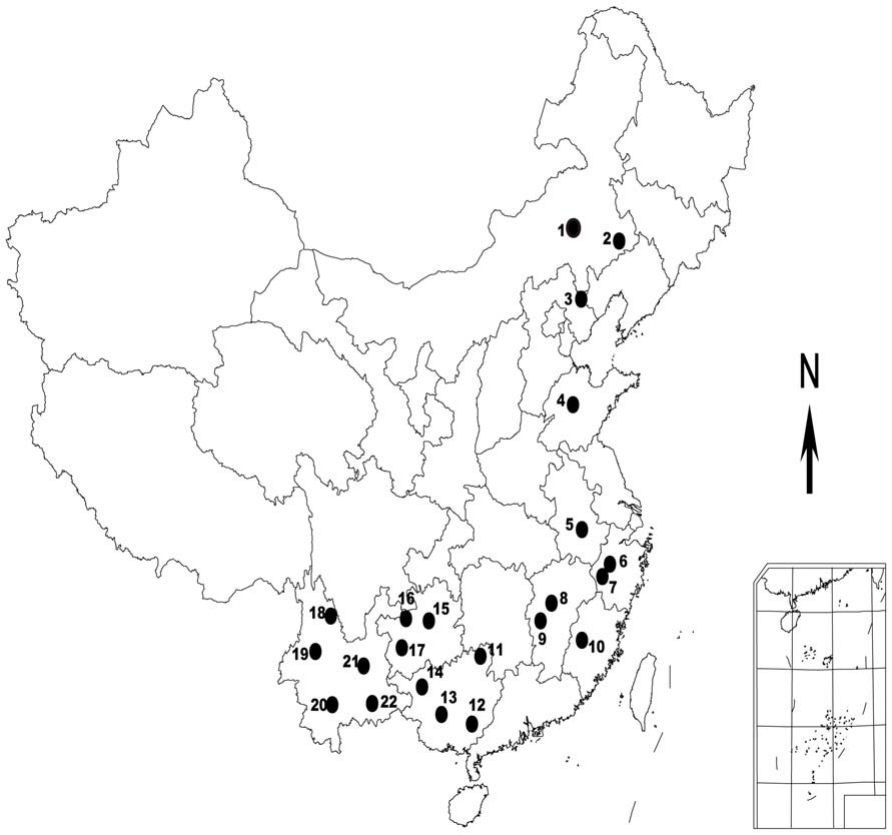
Sampling sites of six closely related *Dendrolimus* pine moth species in China. Detailed geographical information about sampling sites was deposited in Appendix S1.

**Figure 2:**
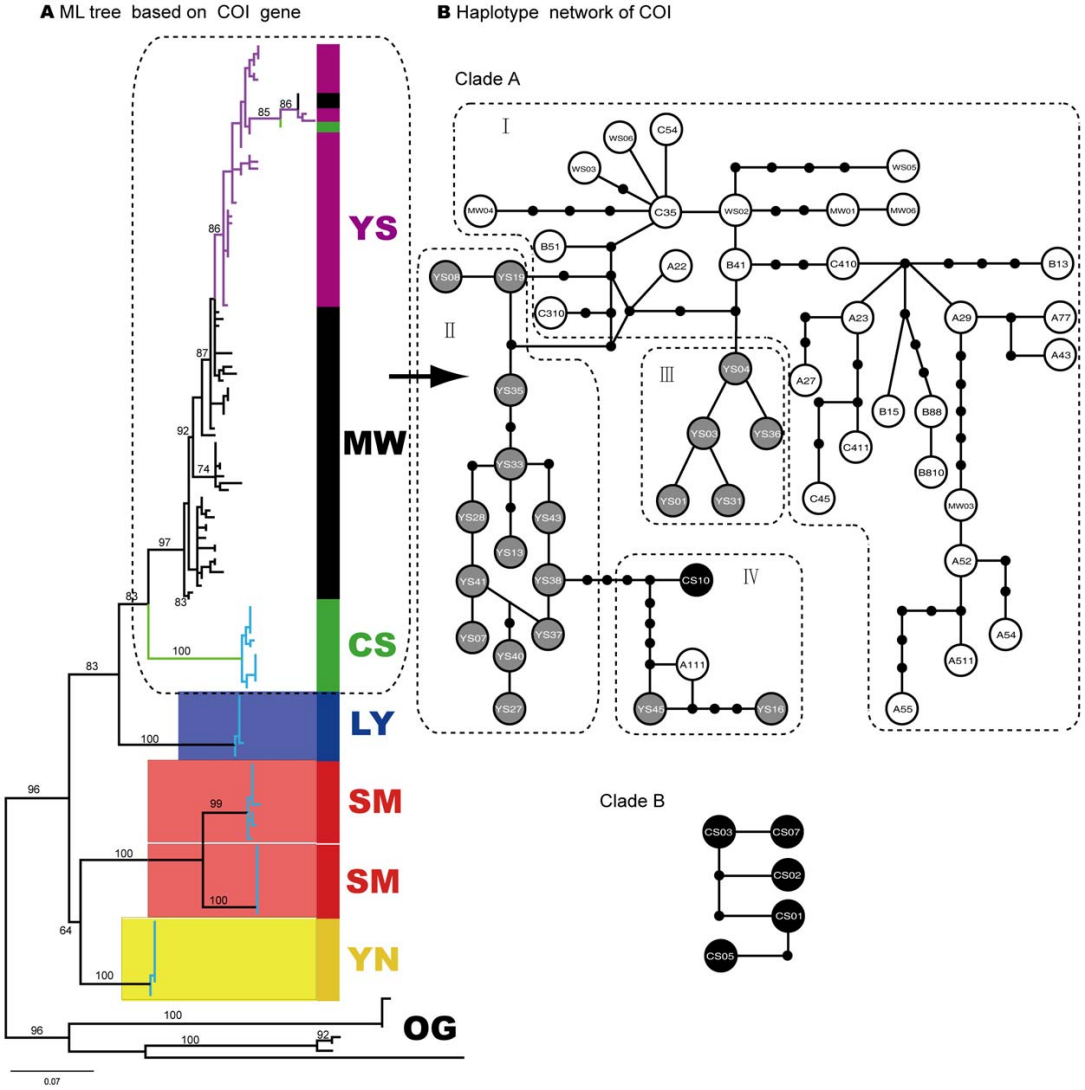
Phylogenetic trees (ML) of six *Dendrolimus* pine moth species constructed with single COI gene and Haplotype network for three mostly closely related species *D.* ** *punctatus*, *D. tabulaeformis*, *D. spectabilis* A) ML tree based on COI gene; Clades with different colors indicate different species respectively. MW - *D. punctatus*, SM - *D. kikuchii*, YN - *D. houi*, YS - *D. tabulaeformis*, CS - *D. spectabilis*, LY - *D. superans*; OG - OUTGROUP; Numbers above branches indicate bootstrap values (less than 50 not shown) (hereinafter). Clades with light blue branches indicate GMYC species, see text for details; B) Haplotype network based on COI gene. Empty circles mean haplotypes of species *D. punctatus*, gray circles indicate haplotypes of species *D. tabulaeformis*, and black circles represent haplotypes of species *D. spectabilis*. Shared haplotypes between different individuals from the same species or different species were listed in Appendix S4, hereinafter.

**Figure 3:**
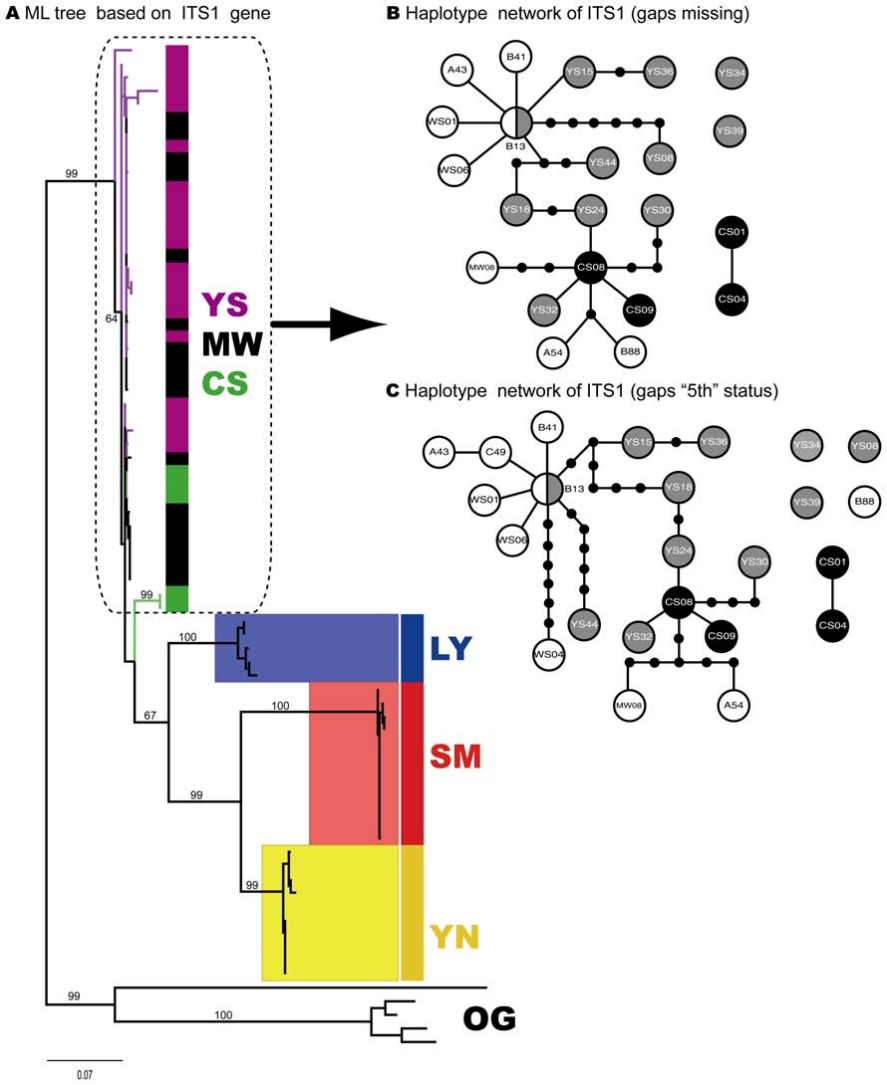
Phylogenetic trees (ML) of six *Dendrolimus* pine moth species constructed with single COI gene and Haplotype network for three mostly closely related species *D.* punctatus, D. tabulaeformis, D. spectabilis. a) ML tree based on ITS1 gene; Clades with different colors indicate different species respectively. MW - *D. punctatus*, SM - *D. kikuchii*, YN - *D. houi*, YS - *D. tabulaeformis*, CS - *D. spectabilis*, LY - *D. superans*; OG - OUTGROUP; Numbers above branches indicate bootstrap values (less than 50 not shown) (hereinafter). Clades with light blue branches indicate GMYC species, see text for details; b) Haplotype network of ITS1 gene (gaps missing); c) Haplotype network of ITS1 gene (gaps “5th” status). Empty circles mean haplotypes of species *D. punctatus*, gray circles indicate haplotypes of species *D. tabulaeformis*, and black circles represent haplotypes of species *D. spectabilis*.

**Figure 4:**
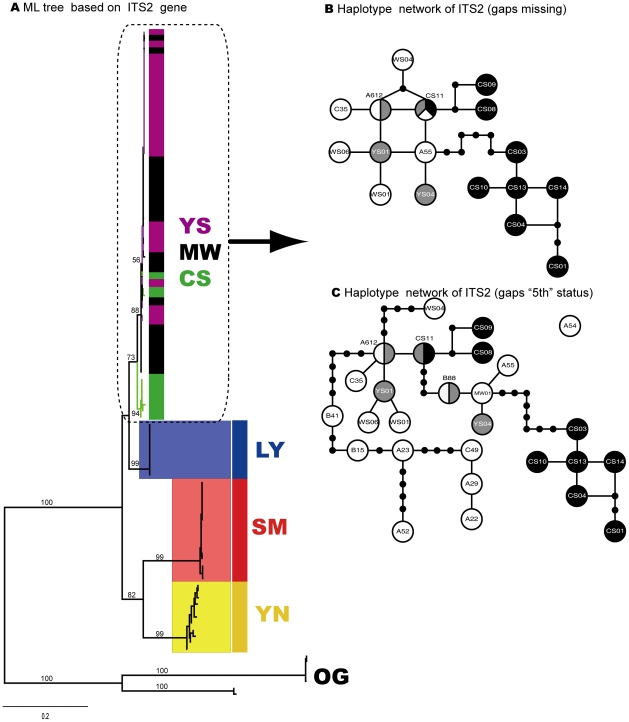
Phylogenetic trees (ML) of six *Dendrolimus* pine moth species constructed with single COI gene and Haplotype network for three mostly closely related species *D.* ** *punctatus*, D. tabulaeformis, *D. spectabilis* A) ML tree based on ITS2 gene; Clades with different colors indicate different species respectively. MW - *D. punctatus*, SM - *D. kikuchii*, YN - *D. houi*, YS - *D. tabulaeformis*, CS - *D. spectabilis*, LY - *D. superans*; OG - OUTGROUP; Numbers above branches indicate bootstrap values (less than 50 not shown) (hereinafter). Clades with light blue branches indicate GMYC species, see text for details; B) Haplotype network of ITS2 gene (gaps missing); C) Haplotype network of ITS2 gene (gaps “5th” status). Empty circles mean haplotypes of species *D. punctatus*, gray circles indicate haplotypes of species *D. tabulaeformis*, and black circles represent haplotypes of species *D. spectabilis*.

**Figure 5:**
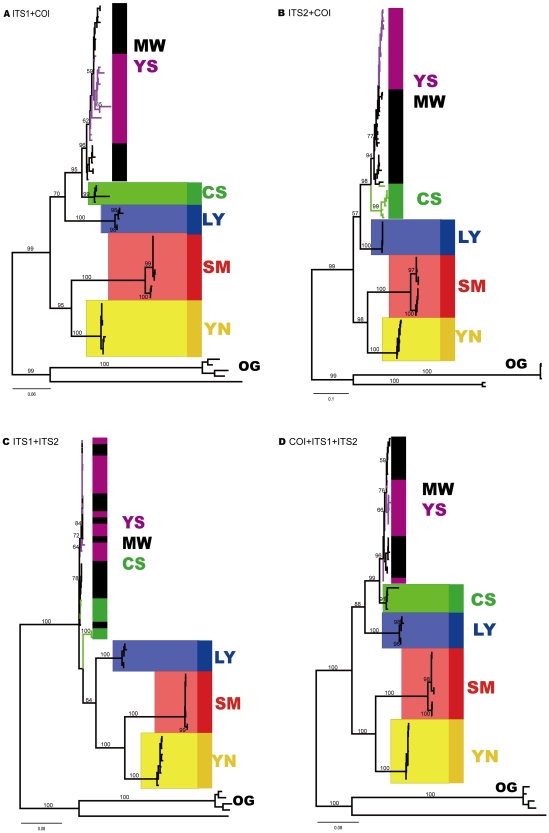
Phylogenetic trees (ML) of six *Dendrolimus* pine moth species constructed with multiple genes (a combination of two or three COI, ITS and ITS2). A) ML tree based on a combination of COI and ITS1 gene; B) ML tree based on a combination of COI and ITS2 gene; C) ML tree based on a combination of ITS1 and ITS2 gene; D) ML tree based on a combination of COI, ITS1, and ITS2 gene.

One hundred and forty specimens from six closely related species of the genus *Dendrolimus* were obtained from 22 sampling locations (Fig. 1; Appendix S1; see Materials and Methods for details). The COI gene achieved the highest sequencing success rate of 100% among the three genes examined, while the other two obtained low success rates, of some 50% (49.30% for ITS1 and 69.30% for ITS2). All sequences successfully sequenced were used in the subsequent alignment analysis. The resultant COI sequence had a length of 652bp, while ITS1 and ITS2 had aligned lengths of 804bp and 656bp respectively. All sequences have been deposited in GenBank with accession numbers JN602739 to JN602878 for COI, JN602879 to JN602947 for ITS1, and JN602948 to JN603044 for ITS2. We obtained seven ML trees, including three single-gene trees based on each of COI, ITS1 and ITS2 genes (Fig. 2, 3, 4, 5; Appendix S4), three gene trees based on the combinations of two of these three genes (COI-ITS1, COI-ITS2, ITS1-ITS2; Fig. 5a–c), and one three-gene tree based on the combination of all three genes (COI-ITS1-ITS2; Fig. 5d). The corresponding NJ trees were provided as online supplementary materials since they presented similar topologies to those ML trees (Appendix S2, S3). Sister group relationship of *D. kikuchii* [55] and *D. houi* [56] was recovered by all single gene, and two-gene and three-gene trees (Fig. 2, 3, 4, 5a). Meanwhile, species level monophyly for *D. superans* [42], *D. kikuchii* and *D. houi* was also found by all these phylogenetic trees from single-gene phylogeny to multiple-gene trees (Fig. 2, 3, 4, 5a). A topology of (((*D. tabulaeformis*, *D. punctatus*, *D. spectabilis*), *D. superans*), (*D. kikuchii*, *D. houi*)) was supported by the COI gene and ITS2 genes repectively, and ((*D. tabulaeformis*, *D. punctatus*, *D. spectabilis*), (*D. superans*, (*D. kikuchii*, *D. houi*)) was supported by the ITS1 gene, while the former was also supported by the three-gene tree (Fig. 5d). The three-gene tree recovered one additional monophyletic clade for species *D. spectabilis* (Fig. 5d). Further, the close relationship of *D. tabulaeformis*, *D. punctatus* and *D. spectabilis* was found by both single-gene and multiple-gene trees (Fig. 2, 3, 4, 5).

Further analysis on these mostly closely related species (*D. tabulaeformis*, *D. punctatus*, and *D. spectabilis*) with haplotype networks shed light on phylogenetic/phylogeographic relationship among them (Fig. 2, 3, 4b). The COI haplotype network was divided into two separate clades, Clade A and Clade B (Fig. 2b). The latter contained haplotypes only from species *D. spectabilis*. The former consisted of haplotypes mostly from species *D. punctatus* and *D. tabulaeformis*, with only one exception (CS10), which was from species *D. spectabilis*. Clade A was further divided into four sub-clades: I, II, III and IV. Sub-clade I only consisted of haplotypes from species *D. punctatus* while sub-clade II and III constituted haplotypes from *D. tabulaeformis*. Sub-clade IV is a clade with mixed haplotypes from all the three species (Fig. 2b). However, there are no shared haplotypes among these three species for COI gene. The COI network indicated that *D. tabulaeformis* has a closer relationship with *D. punctatus* than with *D. spectabilis*, by forming a minimum two-step mutations from hyplotypes of *D. punctatus* (Fig. 2b). *D. spectabilis* showed a relatively distant relationship with *D. punctatus* via at least six-step mutations to the haplotypes of *D. punctatus* (sub-clade IV) and a maximum 11-step mutations to clade A (A111, Fig. 2b). ITS1 networks (both gaps as missing and “5th” status) presented larger variation among these three species by forming a few more separated haplotypes (YS34, YS39,CS01-CS04; YS34, YS08, YS39, B88, CS01-CS04) with 11-step mutations from the main clade (Fig. 3bc). One shared haplotype (B13) between species *D. tabulaeformis* and *D. punctatus* was found, indicating close relationship between these two species. Obviously, treating gaps as “5th” status made the variation among haplotypes become larger than as missing (Fig. 3bc), e.g., haplotype YS08 presented seven-step mutations from the haplotype B13 when gaps were treated as missing, while haplotype YS08 became separated from haplotype B13 with 11-step mutations when gaps were treated as “5th” states. ITS2 networks illustrated that most *D. spectabilis* haplotypes presented 2–7 step mutations (gaps as missing, except haplotype CS11) and 2–9 step mutations from haplotypes of *D. punctatus* and *D. tabulaeformis* (gaps as “5th” status; except CS11) (Fig. 4bc). Haplotypes from *D. tabulaeformis* and *D. punctatus* showed mixed patterns on both networks, indicating a close phylogenetic relationship between these two species (Fig. 4bc). One two-species shared haplotype (A612, between species *D. punctatus* and *D. spectabilis*) and a three-species shared haplotype (CS11, among *D. punctatus*, *D. tabulaeformis*, and *D. spectabilis*) were found on the ITS2 network with gaps as missing (Fig. 4b). Three two-species shared haplotypes (A612 and B88, between *D. punctatus* and *D. tabulaeformis*, and CS11, between *D. tabulaeformis* and *D. spectabilis*) were found when gaps were treated as “5th” states (Fig. 4c).

It is reasonable to assume that the success of species assignment may be higher where the reconstructed evolution of the gene reflects the speciation events, particularly where closely related species are under study [57]. For the individual ML gene trees, we find the GMYC model had no improved fit over the null model. However, since three species (the colored clades in Fig. 2, plus *D. spectabilis* in COI) formed robust monophyletic clades, the GMYC analyses was repeated on a tree in which only sequences belonging to these species of interest were retained. In the case of the ITS loci, we found no significant GMYC clusters. For the COI tree, the GMYC was an improvement over the null model, and was clustered into five ML entities (p = 0.0014, likelihood ratio = 15.6)(Fig. 2a). Interestingly, the COI GMYC groups did not precisely correspond to assigned morphospecies, as *D. kikuchii* was recovered as two separate MOTUs, although this was perhaps not surprising given the relatively long branches (apparent in Fig. 2a) separating the two *D. kikuchii* subclades.

### Mantel Test

**Figure 6:**
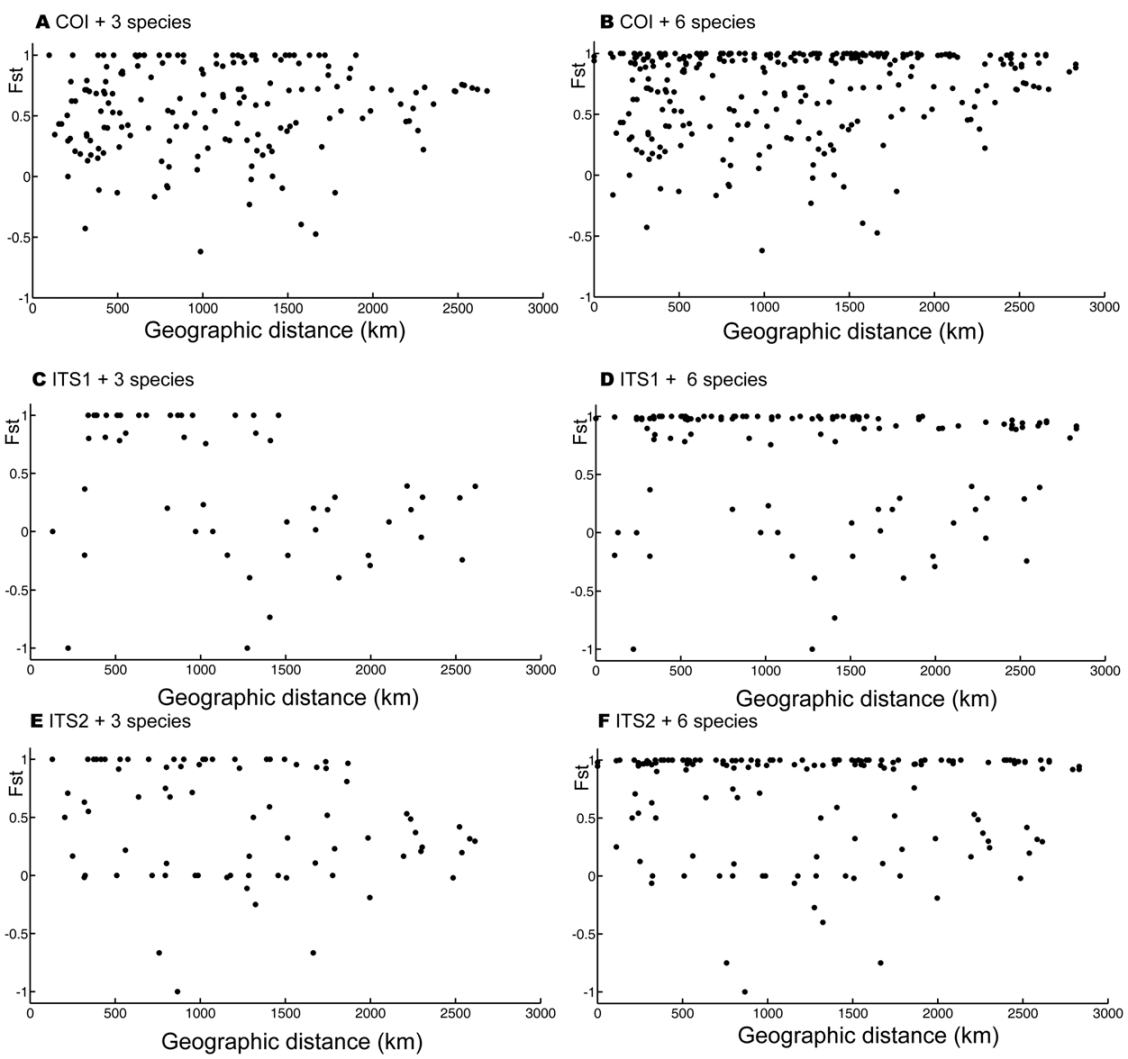
Correlation between Fst and geographical distance with Mantel tests for three mostly closely related species and for all six species with different genes. A) Correlation between Fsts and geographical distances with COI gene for three mostly closely related species *D. punctatus*, *D. tabulaeformis*, *D. spectabilis* (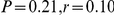); B) Correlation between Fst and geographical distance with COI gene for six species for six species (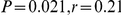 for six species (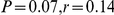); C) Correlation between Fst and geographical distance with ITS1 gene for three mostly closely related species *D. punctatus*, *D. tabulaeformis*, *D. spectabilis* (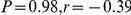); D) Correlation between Fst and geographical distance with ITS1 gene for six species (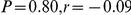); E) Correlation between Fst and geographical distance with ITS2 gene for three mostly closely related species *D. punctatus*, *D. tabulaeformis*, *D. spectabilis* (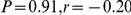); F) Correlation between Fst and geographical distance with ITS2 gene for six species (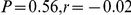).

There was no significant correlation between genetic variation and geographical distances found with each of three genes for the most closely related species (*D. punctatus*, *D. tabulaeformis*, and *D. spectabilis*) (Fig. 6) (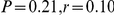 for COI gene; 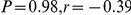 for ITS1 gene; 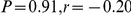 for ITS2 gene). The average Fst values ranged from 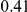 to 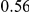 (0.56 for COI; 0.41 for ITS1; 0.47 for ITS2), while mean geographical distances were in the range of 1079.71 to 1161.09 km. The results indicated that the genetic variation among these closely related species did not result from isolation by geographical distance. Some other factors, such as variation in host use, may play important role in the genetic differentiation of these species. Additional Mantel tests on six morphospecies with different genes generally showed no correlation between geographical distance and genetic variation (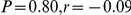 for ITS1 gene; 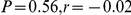 for ITS2 gene; 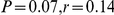 for COI gene).

### Species Assignments with Distance-based Methods and the Neural Network Approach

**Figure 7:**
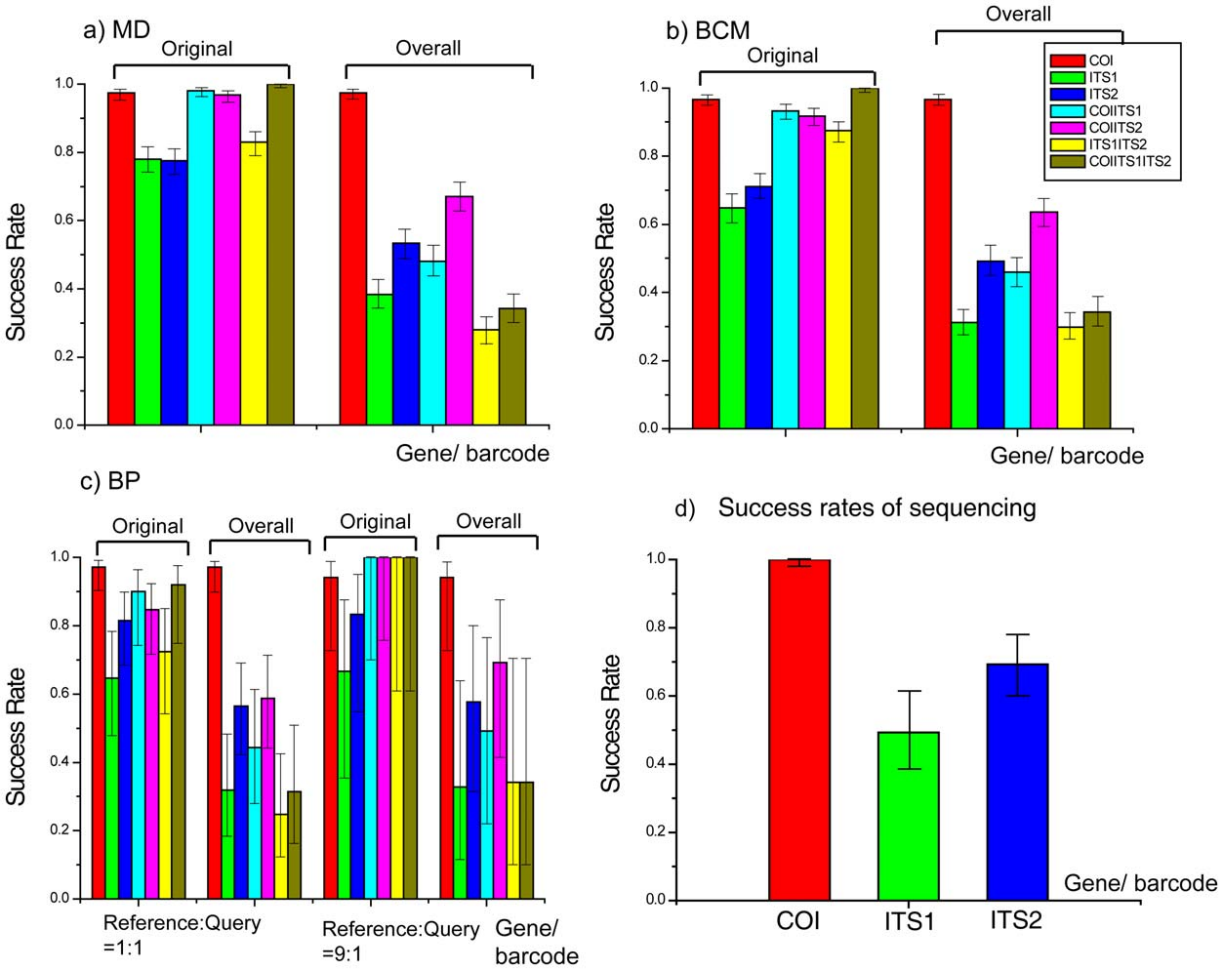
Success rates of species identification based on different gene/barcodes or their combinations for six closely related *Dendrolimus* pine moth species with distance-based methods and neural network approach [**52**]. a) Success rates with MD method [53] based on 500 replications; b) Success rates with BCM method [20] based on 500 replications; c) Success rates with BP-based method [12]; d) Success rates of sequencing for COI, ITS1, and ITS2 genes. Bars with different colors denote different genes/barcodes or their combinations. Vertical solid line with two horizontal short lines indicate 95% confidence intervals of success rates. Bars under “original” and “Overall” mean original success rates and the overall success rates corrected by sequencing success rates respectively (see text for details).

In the case of identification with the MD method, and regardless of the effect of sequencing on success rate, the COI barcode correctly identified 487 individuals from 500 random queries, generating a 97.4% success rate of species identification with 95% confidence interval (CI) (95.60–98.47%), while both the ITS1 and ITS2 barcodes obtained significantly lower species identification success rates of 78.00% (95%CI: 74.16–81.41%) and 77.60% (95%CI: 73.74–81.04%; Fig. 7a). For two-gene barcodes, both COI-ITS1 and COI-ITS2 combinations generated higher species identification success rates (98% with 95% CI: 96.36–98.81% for COI-ITS1, 96.80% with 95% CI: 94.87–98.02%) than that of single gene barcode (ITS1 and ITS2), except for COI. However, the combination of ITS1 and ITS2 (ITS1-ITS2) produced a lower success rate (83.00% with 95% CI: 79.46–86.04%) than even that of the single COI barcode (97.40% with 95% CI: 95.60–98.47%). The ITS1-ITS2 barcode generated slightly higher success rate (83.00%) compared with that of each of them (78.00%, 77.60%), but with no statistic significance. The three-gene barcode (COI-ITS1-ITS2) achieved a 100% species identification success rate, outperforming all other barcodes but COI (with no significant difference compared). However, the overall species identification success rates of these barcodes, from a single-gene to the three-gene system (COI being the exception), dramatically dropped to less than 70.00% (in the range of 29.90–63.60%; Fig. 6a) when taking success rate of sequencing into account. There is no difference in species identification success rates for ITS1 and ITS2, but if the overall identification success rates were considered, ITS1 is better than ITS2, significantly, even both genes obtained lower success rates.

In the case of the BCM method (Fig. 7b), performances of different barcode systems, from single-gene system to three-gene system, presented quite a similar pattern to the MD method. The single COI barcode achieved a success rate of 96.60% (95% CI: 94.62–97.87%) over 500 random queries, significantly outperforming both ITS barcodes (ITS1, ITS2) with a 64.80% success rate with 95% CI: 60.52–68.86% for ITS1, and 71.00% success with 95% CI: 66.87–74.81% for ITS2. The performance of ITS2 was slightly better than that of ITS1, but without statistical significance. The two-gene barcodes (COI-ITS1, COI-ITS2, ITS1-ITS2) significantly outperformed single ITS barcode (ITS1 or ITS2) (COI-ITS1: 93.20%; COI-ITS2: 91.80%; ITS1-ITS2: 87.40%; ITS1: 64.80%; ITS2: 71.00%; Fig. 7b), but to a lesser degree than that of the single COI barcode. The tree-gene barcode system (COI-ITS1-ITS2) achieved the highest success rate of 100% (Fig. 7b). However, if taking efficiency of sequencing into account, the overall species identification success rate of these barcodes abruptly fell to less than 65% (from 29.90 to 63.60%; Fig. 7b), apart from the COI barcode (achieved a success rate of 96.60%). Where sequencing efficiency is considered, the three-gene system obtained an extremely low overall success rate of 34.20%, primarily due to the low sequencing efficiency of ITS (Fig. 7b).

Instead of using the leave-one-out simulation for MD and BCM methods as mentioned above, we used randomly selected reference and query sequences [12] to investigate the performance of different barcode systems. This strategy was employed due to the slow training process which hinders the utility in large scale simulation studies of the BP-based method. Where the ratio of 50% reference sequences to 50% query sequences was used, the COI barcode successfully identified 69 sequences from the randomly chosen set of 71 queries, generating a success rate of 97.2% (95% CI: 90.32–99.23%; Fig. 7c). Both ITS1 and ITS2 produced low success rates of 64.70% (95% CI: 47.90–78.50%), and 81.60% (95% CI: 68.60–90.00%) respectively. The two-gene barcodes (COI-ITS1, COI-ITS2) generated slightly higher success rates (90.00%, 84.80%) than those of ITS barcodes (64.70%, and 81.60%) with no statistic significance, but significantly lower than that of COI barcode (97.20%). The remaining two-gene system (ITS1-ITS2) presented much low species identification success rate compared to those of the above mentioned two (Fig. 7c). The three-gene barcode (COI-ITS1-ITS2) obtained a success rate of 92.00% (95% CI: 75.03–97.78%), which was lower than that of single COI barcode (Fig. 7c). The overall species identification success rates when considering sequencing efficiency, were much lower than those of their corresponding counterparts, but the COI barcode which still achieved a high success rate of species identification (97.20% with 95.00% CI: 90.32–99.23%). Increasing the reference sequences (ratio = 9:1) basically improved species identification success rate for most of these barcodes and their combinations, except for COI barcode (94.10% with 95.00% CI: 72.99-98.95% for the case of reference:query = 1∶1; 97.20% with 95% CI: 90.32–99.23% for the case of reference:query = 9∶1). Considering sequencing success rates, the overall success rates dropped to less than 60.00% (in the range of 24.80–56.50%; Fig. 6c) for the case of 1∶1 reference query ratio, apart from the COI barcode. The success rates of most barcodes and their combinations dropped to less than 70.00% (in the range of 32.80–69.30%, Fig. 6c) for reference:query  =  9:1, but the COI barcode which still obtained a success rate of 94.10% (95% CI: 72.99–98.94%; Fig. 7c). The results of the success rate of sequencing for the three individual genes/barcodes are presented in Fig. 7d. 140 COI PCR products were successfully sequenced, with a 100% sequencing success rate, indicating the reliability of generating the COI barcode, while both ITS1 and ITS2 generated a low sequencing success rate of 49.30% and 69.30% respectively (69/140, 97/140). As a consequence, the barcoding system with one of these two genes generated extremely low overall species identification success rates in most cases.

### Intraspecific, Interspecific Variation, and DNA Barcoding Gaps

The COI barcode obtained an average interspecific K2P distance of 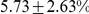, which is about 5 times (4.73) larger than the mean intraspecific distance (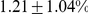, Appendix S5 A) for this closely related pest species group. However, there is no positive DNA barcoding gap for the COI barcode (Appendix S5 A), indicating the difficulty of distinguishing these sibling species. Both ITS1 and ITS2 genes presented greater interspecific genetic variation (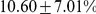 for ITS1; 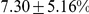 for ITS2) than intraspecific variation (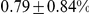 for ITS1;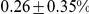 for ITS2). The former is about 13 (13.41) and 28 (28.07) times larger than that of the latter, respectively. Nevertheless, there is still no positive barcoding gaps for these two markers, violating the discrimination of these sibling species (Appendix S5 BC). The multiple-gene barcode system (two or three gene combinations) depicted the same patterns as those of single-gene barcode system (Appendix S5 and S6), further indicating the difficulties in identification for these closely related species. These results are consistent with those of tree-based methods, where species *D. punctatus*, *D. spectabilis*, and *D. tabulaeformis* presented polyphyletic/paraphyletic relationship with each other.

## Discussion

Among six morphspecies, three of them (*D. superans*, *D. kikuchii*, and *D. houi*), were successfully found as monophyletic groups each at the level of species by both single-gene trees and multiple-gene trees (Fig. 2, 3, 4, 5). The phylogenetic relationship among the most closely related species (*D. tabulaeformis*, *D. punctatus* and *D. spectabilis*) was not resolved by the traditional tree-building methods (ML or NJ) with single gene or multiple genes. The three-gene tree found one more monophyletic species clade of *D. spectabilis*, indicating the power of multiple gene markers in discoverying species phylogeny. Further haplotype network analysis for three mostly closely related species indicated that *D. tabulaeformis* has a closer relationship with *D. punctatus* than *D. spectabilis* with *D. punctatus*, although the three morphospecies were even not completely separated on the networks. These results further confirmed their close relationship which may be ascribed to hybridization among them or incomplete lineage sorting. Joint analysis of multiple genes, especially maternal (COI) and bi-parental (nuclear ITS genes here), may suffer from theoretic imperfection since different gene may have different evolutionary history. Therefore, caution should be exercised when combing multiple genes in a phylogenetic analysis. More attention should be paid to the contrasting phylogenetic signals among different genes. Fortunately, there is slight difference in tree topologies between COI gene tree and one of ITS gene trees (ITS1), while ITS2 presents consistent phylogeny with that of COI gene (Fig. 2, 3, 4). Therefore, the combining of these genes was thought to be less problematic in this study. On the other hand, multiple-gene analysis can improve the power for barcoding due to its increasing in genetic diversity.

The segment of the COI gene currently used as the standard barcode for animals is one of the best barcodes among the genes examined in this study, for these closely related pine moth species, regardless of assignment methods. For example, the COI barcode outperformed the other two ITS genes significantly for three non-tree based methods, with good statistical features for species identification success rate. The COI barcode achieved a high success rate of 94.10–97.40% while ITS1 and ITS2 obtained a success rate of 64.70–81.60%. The latter two ITS genes presented slightly different species identification success rates but without statistical significance. The COI barcode outperforms the ITS genes also in terms of its high success rate of sequencing. A hundred percent success rate were achieved for COI region, but a 49.30% success rate for ITS1, and a 69.30% success rate for ITS2 were obtained, although subcloning of these two genes may yield the sequences, it generates inconveniences in a DNA barcoding framework. Non-coding ITS markers, in theory, are expected to be more polymorphic than COI due to suffering from less selection pressure compared to protein-coding COI genes. Therefore, ideally, ITS markers are more suitable for phylogenetic relationship at a lower level, i.e. closely related species. As expected, both ITS1 and ITS2 genes demonstrated larger genetic variations for three relatively distantly related species (*D. superans*, *D. kikuchii*, and *D. houi*) than COI. However, ITS markers become less variable for the most closely related species group (*D. tabulaeformis*, *D. punctatus*, and *D. spectabilis*), mainly because indels/gaps are generally treated as “missing” during the calculation of genetic distances due to the fact that so far no molecular evolutionary models are able to simulate evolution of indels. Furthermore, treating the gaps/indels as missing data may have different effects on the topologies between the most closely related species and the distant related species group. In additional network analysis gaps were treated as “5th” states in the alignments of ITS markers, in order to extract more information from these regions. The low success rate of sequencing for ITS genes may be ascribed to its heterogeneity, which is one of the more problematic issues for the use of ITS2 as DNA barcode. The problems caused by heterogeneity are not limited to its use in DNA barcoding, but for phylogenetic analysis in general [58]. Identifying heterogeneity using measures, such as subcloning, can be applied for use in phylogenetic studies. However, this clearly burdens the DNA barcoding process, as mentioned above. Some successes were reported with ITS barcodes for plants [31]–[32]. However, these studies only used ITS2 sequences that were successfully sequenced, disregarding the sequencing success rates, since most of these data were downloaded from GenBank directly, where only successfully sequenced sequences are deposited. We found that in our pine moth case, sequencing success rates of both ITS1 and ITS2 were low (49.30% for ITS1, 69.30% for ITS2) compared with that of standard COI barcode (100%). Taking sequencing success rates into account, both ITS1 and ITS2 will generate extremely low overall species identification success rates, indicating that ITS genes may be less suitable for DNA barcoding of animals, despite their reported successes in plant. The failure of sequencing for these region resulted from heterogeneity, which indicates the model of concerted evolution [59]–[60] may not be sufficient for the evolution of ITS genes in these closely related pine moth species. Introgression (due to hybridization) and incomplete lineage sorting, or an origin of parapatric species pairs by recent speciation, are all processes that may result in heterogeneity. Our current dataset does not distinguish between these two causes, but further research into this question would be required to understand the process.

Multiple-gene barcoding system achieved better species identification success rates only when each gene possesses a 100% sequencing success rate, otherwise the overall species identification success rate will drop dramatically, at least in our pine moth case. In this study, we firstly proposed the use of overall DNA barcode success rate taking both assignment success and sequencing success into account. The latter has been largely neglected in current DNA barcoding studies. Therefore, the actual species identification success rates were overestimated in some current barcoding studies (e.g. [61]). In our pine moth case, the overall species identification success rates were significantly lower than those of their corresponding species assignment success rates (treated as species identification success rates in current studies). This was the case for both multiple-gene barcoding system and the single-gene barcoding system (except COI), e.g., the three-gene system (COI-ITS1-ITS2) achieved a 100% assignment success rate, but the overall species identification rate is only 34.20%. In addition, the non-tree based DNA barcoding analysis illustrated an advantage over tree-based methods by presenting explicit success rates with statistic testing. The tree-based methods presented only successfully sequenced DNA sequences on a phylogenetic tree.

Mis-assignments only occurred among the three pine moths species, *D. punctatus*, *D. tabulaeformis*, and *D. spectabilis*, whose distribution areas are slightly overlapped [33]–[39]. Both tree- and non-tree based methods provided consistent results, in the formation of a paraphyletic/polyphyletic clade of these three species for the former, or by mis-assigning queries into one of these three species for the latter. The outcome of the tree-based three-gene system was improved by clustering one more additional monophyletic clade species *D. spectabilis*, and the non-tree based three-gene system also achieved a hundred percent success rate without considering sequencing rates. These three monophyletic species did present a complex species status historically [33]–[39]. Our results indicate that the most closely related species *D. punctatus*, *D. tabulaeformis*, and *D. spectabilis* may be still in the process of imcomplete lineage sorting, and occasional hibridizations occurr among them.

## Materials and Methods

### Sampling, DNA Extraction, PCR and Sequencing

One hundred and forty specimens from six closely related species of the genus *Dendrolimus* were sampled from 22 sampling locations (Fig. 1; Appendix S1), throughout their distribution area in China [No specific permits were required for the described field studies, the locations are not privately-owned or protected in any way, and the field studies did not involve endangered or protected species]. Species from the family Liparidae and *Callimorpha principalis* [62] were included as outgroup taxa when constructing phylogenetic trees. DNA samples were prepared from individual insects by extraction of total DNA frozen or 100% ethanol preserved animals. Genomic DNA was extracted using BIOMED DNeasy kit. The COI gene was amplified via PCR using rTaq (TAKARA) with the primers LCO1490 (GGTCA ACAAA TCATAA AGATA TTGG), and HCO2198 (TAAAC TTCAG GGTGA CCAAA AAATCA)[63]. The ITS region of rDNA utilized the primers 18SF1(TACAC ACCGC CCGTC GCTAC TA) and 5.8SB1d(ATGTG CGTTC RAAAT GTCGA TGTTCA) for ITS1, and 5.8SFc(TGAAC ATCGA CATTT YGAAC GCACAT) and 28SB1d(TTCTT TTCCT CCSCT TAYTR ATATG CTTAA) for ITS2 [64]. The amplification reaction was performed in a total volume of 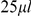, including 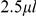 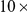
buffer, 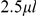 2.5 mM MgCl2, 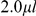 2.5 mM dNTP, 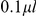 of each primer (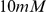), 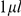 of template DNA, and 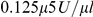 of DNA Taq polymerase, and 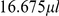 of distilled water. The PCR conditions for the COI gene were as following: 94°C for 2 minutes, 40 cycles of 94°C for 20 seconds, 54°C for 20 seconds, 72°C for 45 seconds, and a final extension at 72°C for 10 minutes. The PCR conditions for ITS region were: 94°C for 2 min, 40 cycles of 94°C for 20 seconds, 51°C(ITS1) and 35°C(ITS2) for 30 seconds, 72°C for 15 seconds, and a final extension at 72°C for 10 minutes. Sequencing was performed with an ABI3130 sequencer.

### Processing of DNA Sequences

The raw DNA sequences were all checked manually by eye. After trimming the ends of the raw sequences, they were aligned using MUSCLE [65] under default parameters. Besides single-gene datasets (COI, ITS1, ITS2), we also assembled three two-gene (COI-ITS1, COI-ITS2, ITS1-ITS2) datasets and a single three-gene data set (COI-ITS1-ITS2), named as two-gene barcoding system and three-gene barcoding system hereinafter.

### Maximum Likelihood Inferences and Neighbor-joining Reconstruction of Species Phylogeny with Single and Multiple Genes

To explore phylogenetic relationship among these closely related species, we constructed Maximum likelihood trees (ML) for these species with each single gene, and their combinations via the fast ML program PHYML3.0 [66]. Initially, NNIs search was used to have a rough idea of the phylogeny. Secondly, a SPR search was performed to generate the final ML tree. K2P model was used as the model of nucleotide substitution [1]–[2]. Nucleotide frequencies, the transition/transversion ratio, and proportion of invariable sites were all estimated in the maximum likelihood framework by the program. Branch supports were estimated using 1000 bootstrap replications. All other parameters were set as default settings. Additionally, we constructed a neighbor-joining tree (NJ, [67]) for each dataset. NJ trees were built using MEGA4.0 [68] with a K2P molecular evolutionary model [1]–[2]. Successful identification was inferred where sequences from the same species formed a monophyletic group although treating reciprocal monophyly as species identification success remains controversial [45]. We next determined whether the individual gene trees formed monophyletic groups possessing branching characteristics of species (a reduced within-group branching rate), and whether these delineated groups corresponded to the morphospecies. The ML trees were delimited into operational taxonomic units using the generalized mixed Yule coalescent approach (GMYC), which integrates both within species (coalescent) and between species (Yule) branching characteristics, finding the most likely position in which a shift between the two has occurred. The tree was dereplicated by identifying and pruning terminals with no molecular divergence from their neighbours, and an ultrametric tree generated by non-parametric rate smoothing (as implemented in r8s, [69]–[70]), upon which we apply the single threshold GMYC model [71]. The groups delimited thus are compared to a null model of a single coalescent group.

### Network Analysis and Mantel Tests

Traditional bifurcating trees are less powerful to resolve relationship among intraspecific populations and closely related species, while haplotype networks can provide significant inferences about evolutionary relationships among them [72]–[74]. Therefore, we constructed haplotype networks for the most closely related species, *D. punctatus, D. tabulaeformis*, and *D. spectabilis*, with each gene marker (COI, ITS1 and ITS2). For the latter two genes, gaps in the alignments were treated as “missing” or “5th” states respectively. To test whether geographically closer species/populations tend to be genetically more similar, correlation between geographical distance and Fst were performed with Mantel test (1000 permutations) implemented in Arlequin 3.1 [75]. Furthermore, Mantel tests were performed at two different scales: one was within the most closely related species (*D. punctatus*, *D. tabulaeformis* and *D. spectabilis*), another was for all six morphospecies although the Mantel test is generally performed at the species level for phylogeographic aims. For the latter analysis, we only wanted to investigate the phylogeographic relationship among these six morphospecies on a longer time span.

### Species Assignments with Distance-based Methods and the Neural Network Approach

Distance-based methods of species assignments in conjunction with computer simulations are capable of determining statistical significance of species identification success rates. We therefore performed the “best close match” (BCM) ([20]), and a minimum distance (MD) method, utilizing “single-sequence-ommission” or “leave-one-out” simulation. In these simulations, we remove one sequence at a time and use it as a query, with all other sequences remaining as the reference database. We performed 500 random replications for each dataset. The “best close match” (BCM, [20]) identification protocol first identifies the best barcode match of a query, but only assigns the species name of that barcode to the query if the barcode is sufficiently similar. This approach requires a threshold similarity value that defines how similar a barcode match needs to be before it can be identified. Such a value could be estimated for a given data set by obtaining a frequency distribution of all intraspecific pairwise distances and determining the threshold distance below which 95% of all intraspecific distances are found. The “BCM” approach is implemented in the computer program TaxonDNA ([20]). The Minimum Distance (MD) method is implemented in a program package MD [53]. With these distance-based methods, we further examined the efficiencies of each single barcode (COI, ITS1, ITS2), two-gene barcodes (COI-ITS1, COI-ITS2, ITS1-ITS2), and the three-gene barcode (COI-ITS1-ITS2) in success rate of species identification.

BP Neural Network-based (BP-based method or BP) species identification has recently been proposed by Zhang and his colleague [12], [52]. The BP-based method proved to be powerful in species assignments via DNA sequences, especially for closely related species [12]. As mentioned above, we have three single gene datasets, three two-gene datasets and one three-gene dataset. Each of these datasets were randomly divided into a reference dataset and a query dataset respectively. The reference dataset was used to train a BP-Neural Network model, while the query dataset as a test dataset. We considered two scenarios - reference:query = 9∶1, and 1∶1. In the former, nine of ten sequences in the dataset were randomly chosen as reference sequences, whereas in the later, one of two sequences was used as reference sequences. For all these simulations, the learning rate was set to 0.2, moment value 0.5, and training goal 0.00001, as implemented in the program BPSI2.0 [52].

### Success Rate of Species Identification and Confidence Intervals

The success rate of species identification is defined with the following formula [12]:
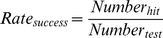
(1)


where 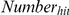 and 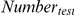 are the numbers of sequences successfully hit by the method under study and the number of total query sequences examined, respectively. A success hit is counted if a query is assigned to its correct species name in the database. Since success rates of sequencing for different genes might affect the final success rate of species assignments, we further define a overall success rate, taking sequencing success into account, measured as in the following equation.
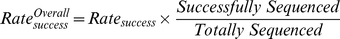
(2)where, 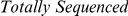 and 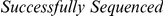 denote the total number of specimens submitted to sequencing, and the number of successfully sequenced for that species.

Binary data indicating the presence (successful identification) or absence (failed identification) of a specific attribute are often modeled as random samples from a Bernoulli distribution with parameter 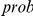, where 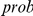 is the proportion in the population with that attribute. A 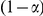-level confidence interval (CI) for 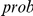 is calculated by the following formula [76]:
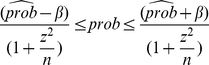
(3)where 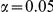, 
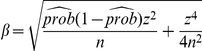
, 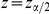 (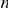 is the number of replications, and 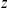 is the critical value corresponding to an area 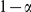 under the standard normal curve).

### Intraspecific, Interspecific Variation, and DNA Barcoding Gaps

The distance between intraspecific and interspecific variation (the DNA barcoding gap), is considered as an important term in DNA barcoding practice. Clearly, a large DNA barcoding gap makes species discrimination possible and easy. Conversely, small or negative DNA barcoding gaps blur species boundaries, and hamper species assignation in DNA barcoding. To search for the reason for failures in species identification, we further explored the intraspecific and interspecific variations within this closely related species group, calculated DNA barcoding gaps for each gene, and for combined two or three - gene barcodes. A Perl script was developed for this task.

## Supporting Information

Appendix S1 **Taxon information, detailed sampling sites, genes used.**
(XLS)

Appendix S2 **Phylogenetic trees (NJ) of six closely related** ***Dendrolimus*** **pine moth species constructed with single gene (COI, ITS1 or ITS2).** a) NJ tree based on COI gene; b) NJ tree based on ITS1 gene; c) NJ tree based on ITS2 gene. Clades with different colors indicate different species respectively. MW - *D. punctatus*, SM - *D. kikuchii*, YN - *D. houi*, YS - *D. tabulaeformis*, CS - *D. spectabilis*, LY - *D. superans*; OG - OUTGROUP; Numbers above branches indicate bootstrap values (less than 50 not shown) (hereinafter).
(TIF)

Appendix S3 **Phylogenetic trees (NJ) of six closely related** ***Dendrolimus*** **pine moth species constructed with multiple genes (a combination of two or three COI, ITS and ITS2).** a) NJ tree based on a combination of COI and ITS1 gene; b) NJ tree based on a combination of COI and ITS2 gene; c) NJ tree based on a combination of ITS1 and ITS2 gene; d) NJ tree based on a combination of COI, ITS1, and ITS2 gene.
(TIF)

Appendix S4 **List of shared haplotypes between different individuals of the same species or different species.**
(XLS)

Appendix S5 **Histograms of intra-(in red) and inter-specific (in blue) pairwise distance between single-gene barcodes for six closely related** ***Dendrolimus*** **pine moth species.**
(TIF)

Appendix S6 **Histograms of intra-(in red) and inter-specific (in blue) pairwise distance between multiple-gene barcodes for six closely related** ***Dendrolimus*** **pine moth species.**
(TIF)
