# Supplementary figures and images for: Phylogenetic Reconstruction and DNA Barcoding for Closely Related Pine Moth Species (Dendrolimus) in China with Multiple Gene Markers
Source: PLoS One. 2012 Apr 3;7(4):e32544. doi: 10.1371/journal.pone.0032544 (PMC3317921; doi:10.1371/journal.pone.0032544)

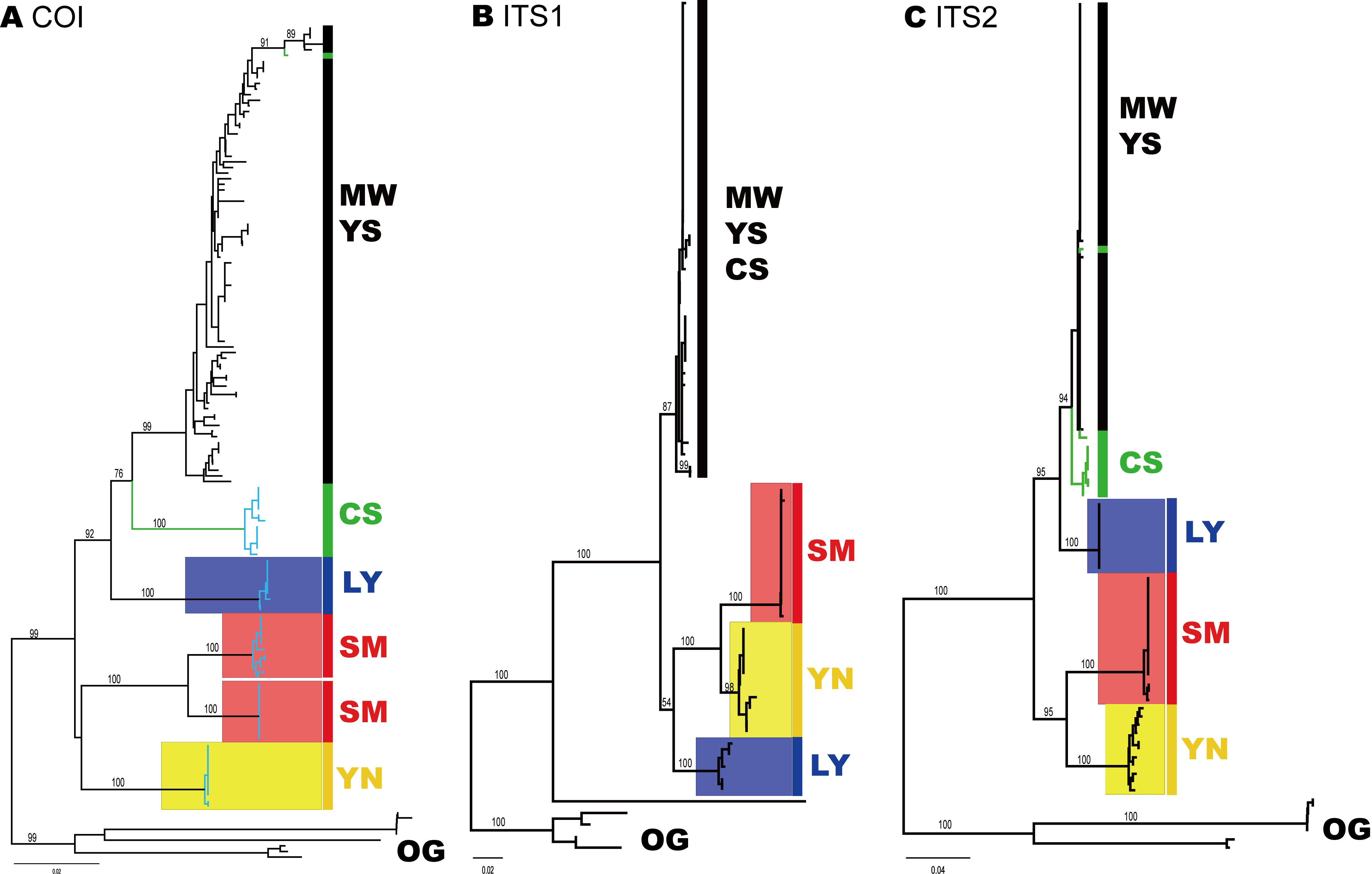

Supplement: Appendix S2 — Phylogenetic trees (NJ) of six closely related Dendrolimus pine moth species constructed with single gene (COI, ITS1 or ITS2). a) NJ tree based on COI gene; b) NJ tree based on ITS1 gene; c) NJ tree based on ITS2 gene. Clades with different colors indicate different species respectively. MW - D. punctatus, SM - D. kikuchii, YN - D. houi, YS - D. tabulaeformis, CS - D. spectabilis, LY - D. superans; OG - OUTGROUP; Numbers above branches indicate bootstrap values (less than 50 not shown) (hereinafter). (TIF) [file pone.0032544.s002.tif]

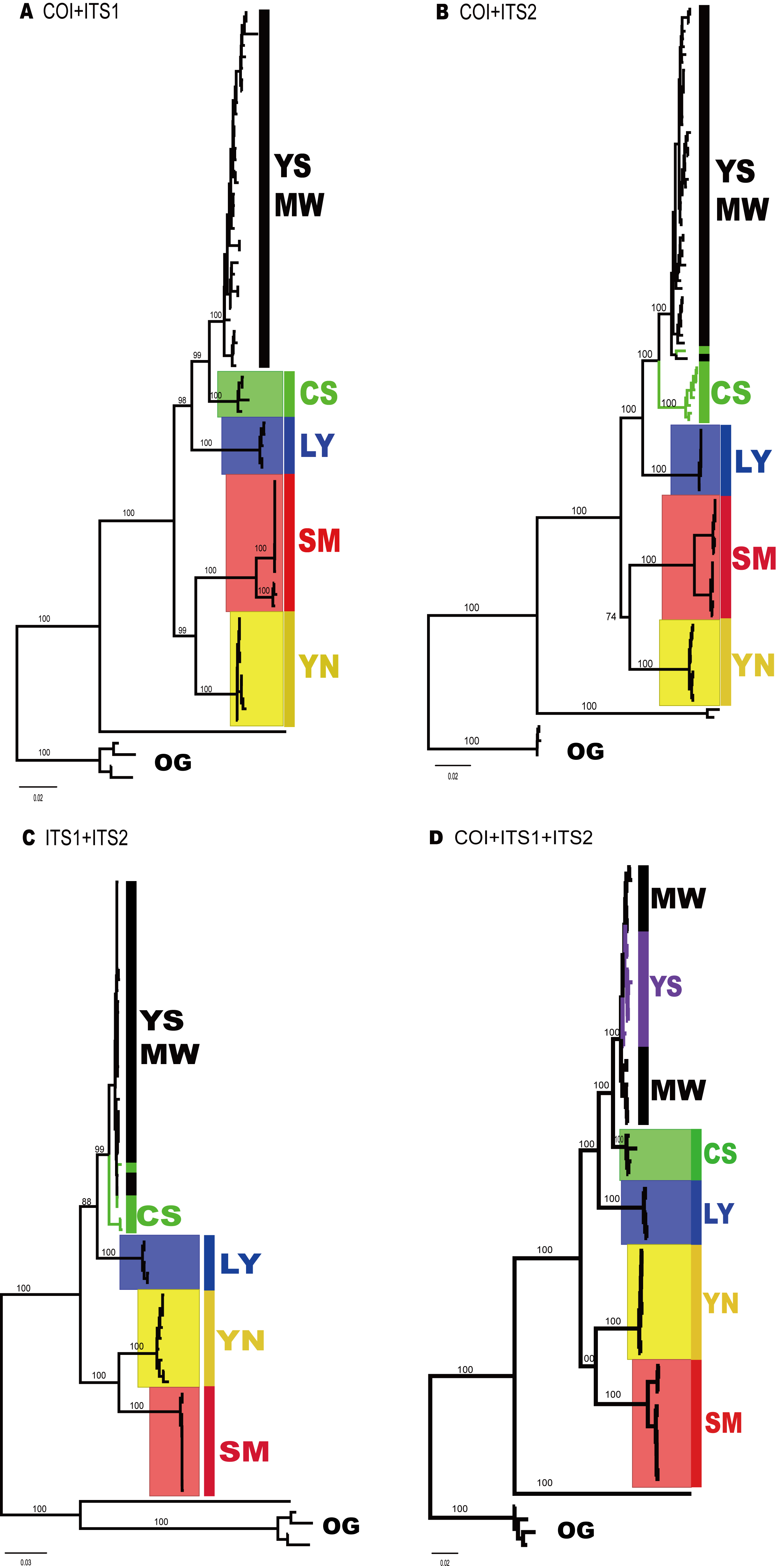

Supplement: Appendix S3 — Phylogenetic trees (NJ) of six closely related Dendrolimus pine moth species constructed with multiple genes (a combination of two or three COI, ITS and ITS2). a) NJ tree based on a combination of COI and ITS1 gene; b) NJ tree based on a combination of COI and ITS2 gene; c) NJ tree based on a combination of ITS1 and ITS2 gene; d) NJ tree based on a combination of COI, ITS1, and ITS2 gene. (TIF) [file pone.0032544.s003.tif]

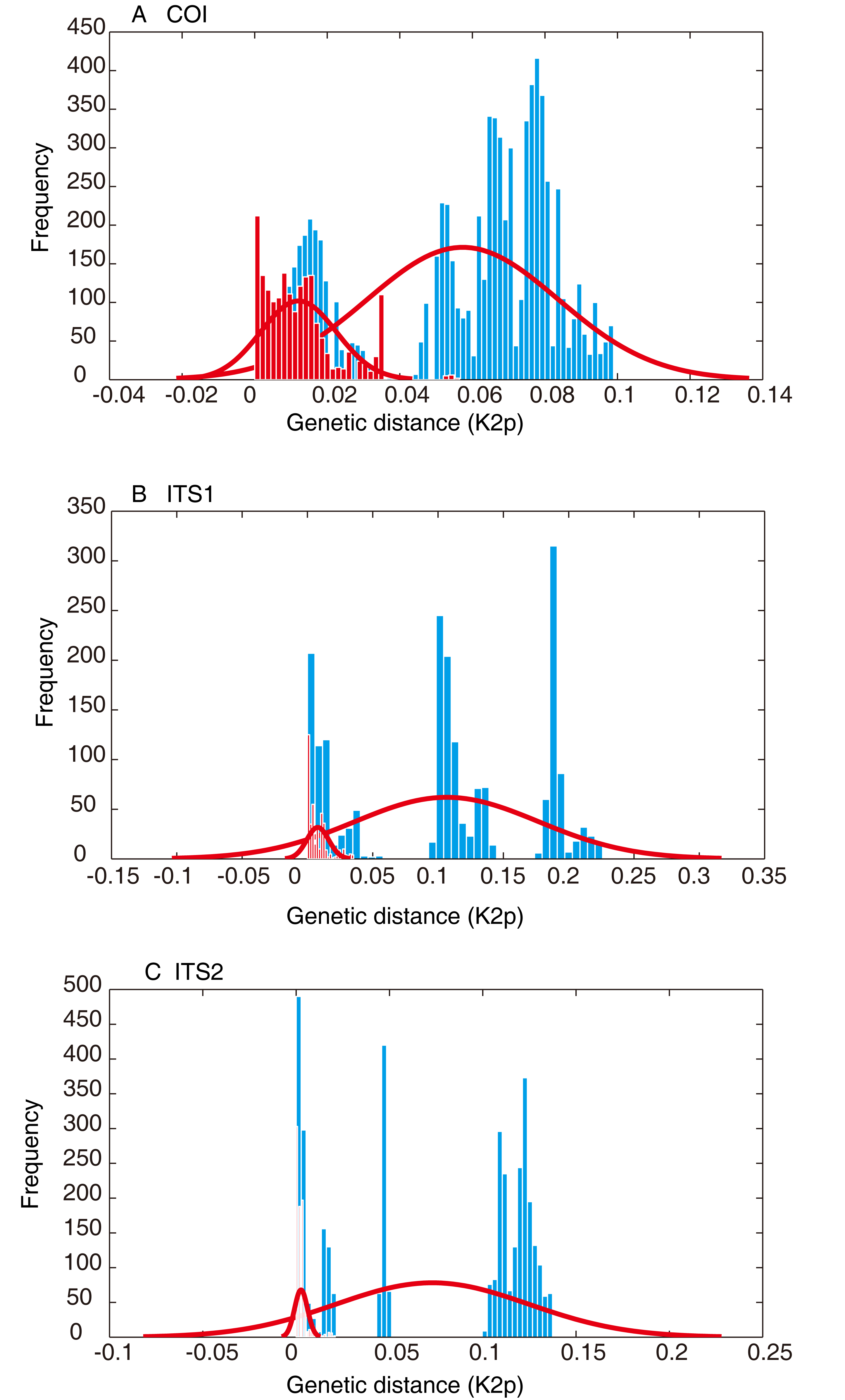

Supplement: Appendix S5 — Histograms of intra-(in red) and inter-specific (in blue) pairwise distance between single-gene barcodes for six closely related Dendrolimus pine moth species. (TIF) [file pone.0032544.s005.tif]

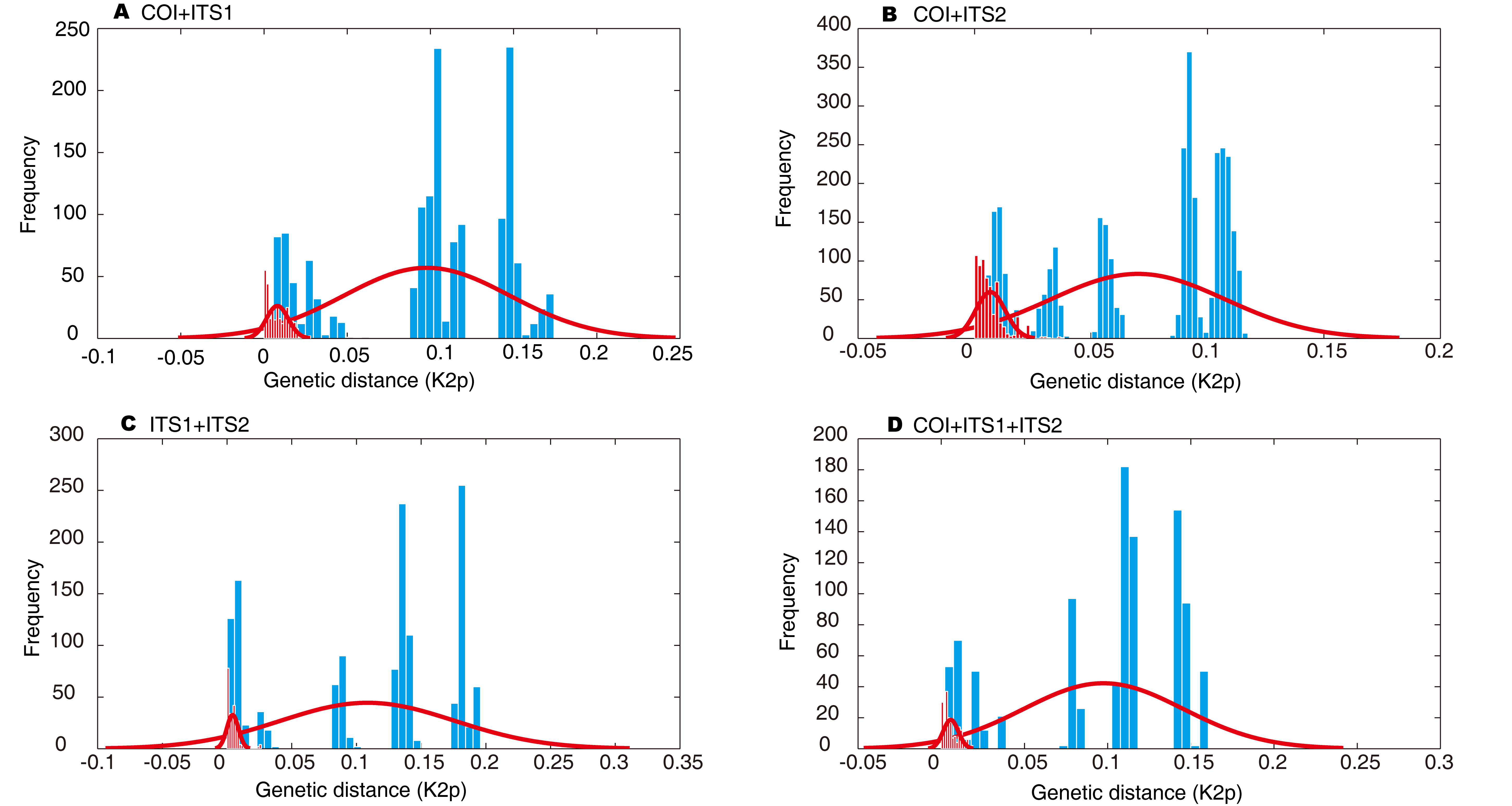

Supplement: Appendix S6 — Histograms of intra-(in red) and inter-specific (in blue) pairwise distance between multiple-gene barcodes for six closely related Dendrolimus pine moth species. (TIF) [file pone.0032544.s006.tif]
